# Expression of Concern: ΔNp73, A Dominant-Negative Inhibitor of Wild-type p53 and TAp73, Is Up-regulated in Human Tumors

**DOI:** 10.1084/jem.2002017906102025e

**Published:** 2025-06-27

Vol. 196, No. 6 | https://doi.org/10.1084/jem.20020179 | September 16, 2002

The authors of this paper notified the *Journal of Experimental Medicine* of an error made during Figure 3 C assembly. The same blot was used for 14-3-3σ and p21 in lanes 6 and 7, making it unclear whether the data represent 14-3-3σ or p21. Due to the time elapsed since publication, the authors regret that they no longer have access to the original data. Consequently, *JEM* and the authors are unable to verify the validity of Figure 3 C. The authors stand by the conclusions of the study.
